# Concurrent Subacute Stent Thrombosis and Left Ventricular Thrombus After STEMI: A Therapeutic Dilemma

**DOI:** 10.1016/j.jaccas.2026.106943

**Published:** 2026-03-11

**Authors:** Juan D. Palomar, Carlos A. Arias, Jeffrey Castellanos, Nelson L. Moreno, David E. Aparicio Martínez, Sergio D. Zabaleta Orozco

**Affiliations:** a Programa de Cardiología-Facultad de Medicina, Fundación Universitaria Sanitas, Bogotá, Colombia; b Departamento de Medicina Interna, Clínica Universitaria Colombia, Clínica Colsanitas S.A., Grupo Keralty, Bogotá, Colombia; c Programa de Falla Cardiaca, Clínica Universitaria Colombia, Clínica Colsanitas S.A., Grupo Keralty, Bogotá, Colombia; d Departamento de Cardiología, Clínica Universitaria Colombia, Clínica Colsanitas S.A., Grupo Keralty, Bogotá, Colombia; e Facultad de Medicina, Universidad Surcolombiana, Neiva, Colombia

**Keywords:** anticoagulation, cardiac MRI, direct oral anticoagulants, left ventricular thrombus, stent thrombosis, triple therapy, STEMI

## Abstract

**Background:**

Left ventricular thrombus (LVT) is a complication of anterior ST-segment elevation myocardial infarction (STEMI) after delayed reperfusion. The coexistence of LVT and stent thrombosis represents a therapeutic challenge with no defined anticoagulation strategy.

**Case Summary:**

A 74-year-old man with anterior STEMI underwent rescue percutaneous coronary intervention with drug-eluting stents to the left anterior descending artery (LAD) and right coronary artery after 12 hours and was discharged on dual antiplatelet therapy. Three weeks later, he re-presented with severe chest pain and persistent ST-segment elevation. Coronary angiography demonstrated LAD stent thrombosis, treated with repeat angioplasty. Subsequent imaging revealed anterior-apical hypokinesia and a 10 × 6 mm apical LVT. Warfarin was added to dual antiplatelet therapy, achieving anticoagulation before discharge.

**Discussion:**

This case highlights limited evidence in the setting of coexisting LAD stent thrombosis and LVT after STEMI. Warfarin remains widely used, while data supporting direct oral anticoagulants are scarce.

**Take-Home Message:**

Individualized antithrombotic strategies balancing ischemic and bleeding risk are essential.

## Visual Summary

**Figure:**
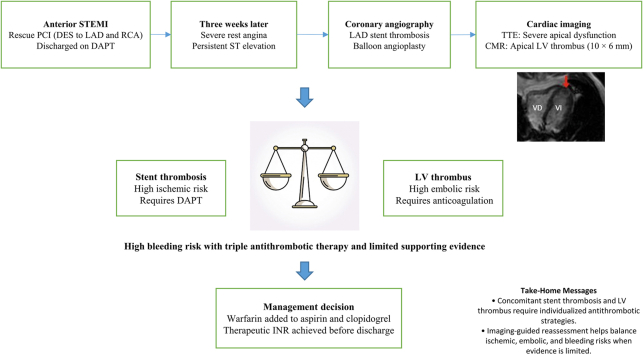

**Visual Summary:**
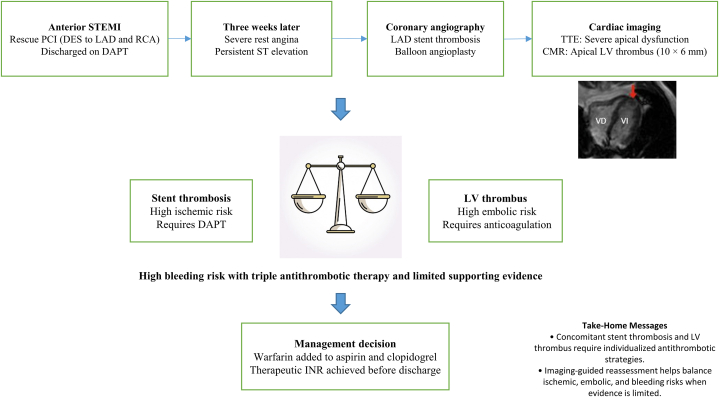
Imaging-Guided Diagnostic and Therapeutic Approach for Concomitant Subacute Coronary Stent Thrombosis and Left Ventricular Thrombus After STEMI CMR = cardiac magnetic resonance; DAPT = dual antiplatelet therapy; DES = drug-eluting stent; INR = international normalized ratio; LAD = left anterior descending artery; LV = left ventricular; PCI = percutaneous coronary intervention; RCA = right coronary artery; STEMI = ST-segment elevation myocardial infarction; TTE = transthoracic echocardiography.

## History of Presentation

A 74-year-old man presented with 30 minutes of severe resting angina associated with dyspnea. He had been discharged 3 weeks earlier after treatment for an anterior ST-segment elevation myocardial infarction (STEMI). On examination, he was hemodynamically stable, with an otherwise unremarkable physical examination.

**Take-Home Messages**

• Severe apical dysfunction after anterior STEMI should prompt early suspicion for left ventricular thrombus, even when initial transthoracic echocardiography is nondiagnostic, with cardiac magnetic resonance imaging playing a pivotal role in confirmation and follow-up.
• In patients with recent PCI and high thrombotic risk, antithrombotic therapy should be individualized using an imaging-guided approach that balances ischemic and bleeding risks rather than fixed, time-based regimens.

## Past Medical History

The patient's medical history was significant for hypertension and type 2 diabetes mellitus. Three weeks prior, he had undergone rescue percutaneous coronary intervention (PCI) with drug-eluting stent implantation in the left anterior descending artery (LAD) and right coronary artery and was discharged on dual antiplatelet therapy with aspirin and clopidogrel.

## Differential Diagnosis

The differential diagnosis included left ventricular aneurysm, post–myocardial infarction pericarditis, coronary vasospasm, acute ischemia-related heart failure, and other mechanical complications of myocardial infarction.

## Investigations

**Figure 1:**
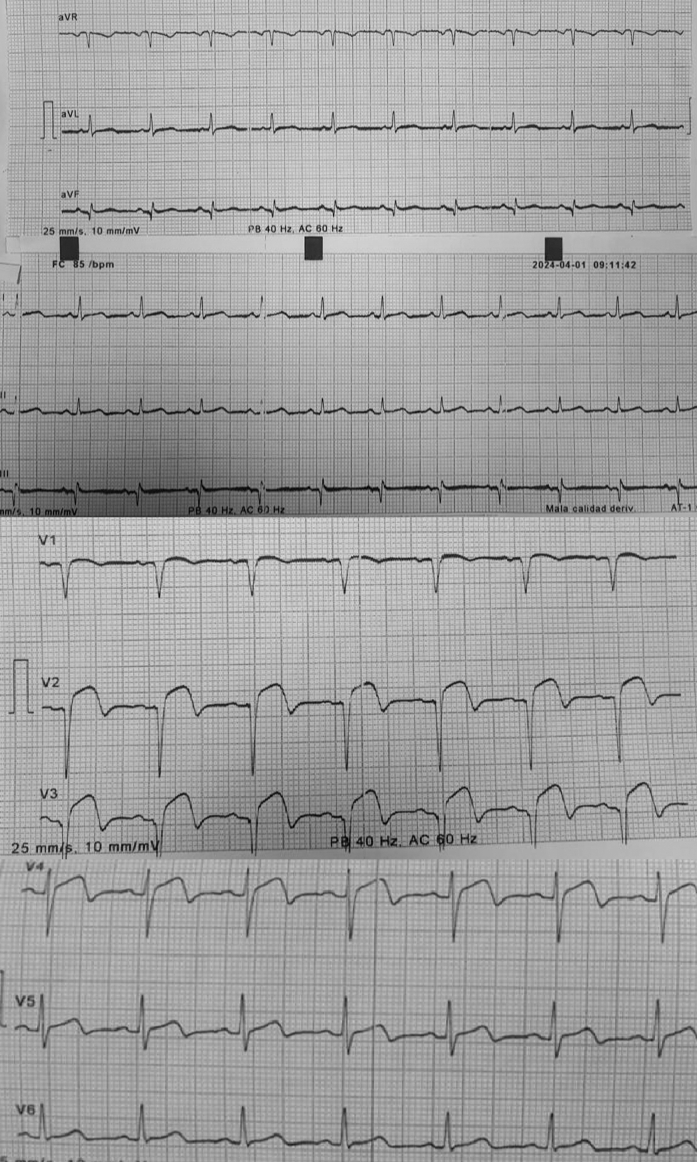
Electrocardiogram During Second Hospitalization Electrocardiogram demonstrating persistent ST-segment elevation in the anterior leads during the second hospitalization.

**Figure 2:**
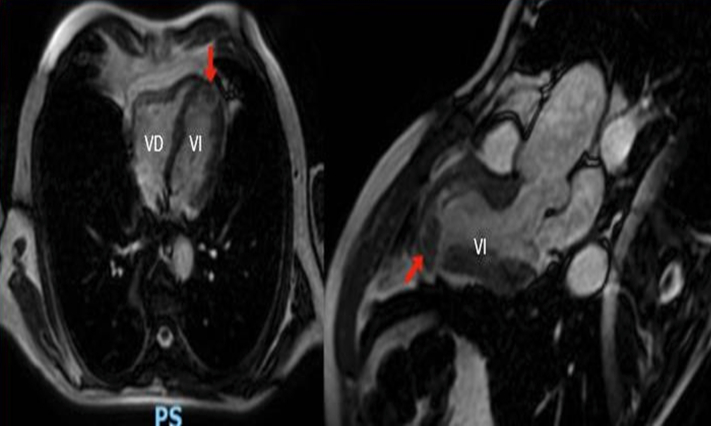
Cardiac Magnetic Resonance 4- and 2-Chamber Views Cardiac magnetic resonance confirming the presence of a 10 × 6 mm intracardiac thrombus in the left ventricle (red arrow), with areas of wall thinning consistent with a prior acute myocardial infarction.

During this second hospitalization, electrocardiography demonstrated persistent ST-segment elevation in leads V2 and V3, which had not been present at discharge (Figure 1). High-sensitivity cardiac troponin T was elevated at 570 ng/L (normal <14 ng/L). At the time of the second presentation, the patient reported adherence to dual antiplatelet therapy. Transthoracic echocardiography (TTE) revealed extensive anteroseptal akinesia and hypokinesia of all apical segments. Repeat coronary angiography confirmed subacute thrombosis of the LAD stent, consistent with myocardial infarction type 4b, and was successfully treated with balloon angioplasty. Subsequent cardiac magnetic resonance (CMR) identified a 10 × 6 mm apical left ventricular thrombus (LVT) (Figure 2).

## Management

Anticoagulation with warfarin was initiated in addition to antiplatelet therapy with aspirin and clopidogrel.

## Outcome and Follow-Up

The hospital course was uncomplicated. After achieving a therapeutic international normalized ratio of 2 to 3, the patient was discharged on triple antithrombotic therapy for 1 month, with planned follow-up in the cardiology and anticoagulation clinics; longer-term outcomes were not yet available at the time of article submission.

## Discussion

The coexistence of coronary stent thrombosis and LVT after STEMI represents a rare but high-risk clinical scenario without a clearly defined antithrombotic strategy. In the present case, the concomitant occurrence of both entities after an extensive anteroseptal transmural infarction required a nonconventional approach to antithrombotic therapy, with careful balancing of competing thrombotic and bleeding risks in the context of limited high-quality evidence to guide management.^1^

Although contemporary PCI has substantially reduced the incidence of the classic complications after transmural infarction, delayed or ineffective reperfusion remains a key determinant of thrombotic sequelae after STEMI. In this patient, several clinical and paraclinical features indicated an elevated thrombotic risk, particularly for LVT formation, while also contributing to intracoronary thrombosis. These included extensive anterior necrosis, persistent ST-segment elevation, severe hypokinesia of apical segments on echocardiography, LAD involvement as the culprit vessel, left ventricular ejection fraction <35%, and failed pharmacologic reperfusion requiring rescue PCI performed 12 hours after symptom onset. These findings are consistent with data from a meta-analysis of more than 2,000 patients in whom LVT was detected by CMR within 1 month after STEMI, demonstrating an overall incidence of 6.3%. The prevalence was substantially higher among patients with anterior infarction and reduced left ventricular ejection fraction (<50%), reaching 19.2%.^2^

TTE remains the initial screening modality for mechanical complications after acute coronary syndromes because of its wide availability and routine use in clinical practice. However, its sensitivity for detecting LVT is limited, with reported variability ranging from 29% to 64% depending on contrast use and timing relative to the index infarction.^3^ In an effort to improve detection, Weinsaft et al^4^ proposed an echocardiographic algorithm incorporating the apical wall motion score, calculated by grading motion abnormalities across 5 apical segments. Threshold values ≥7 without contrast or ≥5 with contrast demonstrated a negative predictive value of 100% for excluding thrombus.^4^ Notably, transesophageal echocardiography did not provide superior diagnostic performance. In the present case, TTE did not initially detect thrombus but revealed profound apical dysfunction, with an apical wall motion score of 10, prompting further evaluation with CMR, which is considered the reference standard for LVT assessment because of its high sensitivity (85%) and specificity (100%).^3^

Given the potentially catastrophic consequences of systemic embolism, anticoagulation is indicated when LVT is diagnosed. Warfarin has traditionally been the most widely used anticoagulant in this setting, with guideline-recommended therapeutic targets corresponding to an international normalized ratio of 2 to 3 for a typical duration of 3 months.^1^^,^^5^ In selected scenarios, extended anticoagulation may be considered based on persistent thrombus mobility or lack of organization on follow-up imaging. However, these recommendations do not adequately address patients who also require dual antiplatelet therapy after recent PCI.^5^

Recent interest has increasingly shifted toward the evaluation of direct oral anticoagulants (DOACs) as alternatives to vitamin K antagonists for the treatment of LVT. Nevertheless, the available evidence remains limited and is derived largely from small, retrospective studies.^6^ The No-LVT trial, a multicenter, randomized, open-label study, enrolled 79 patients with newly diagnosed LVT detected by echocardiography and randomized them to receive either rivaroxaban (n = 39) or warfarin (n = 40).^7^ At the 1-, 2-, and 6-month follow-up, thrombus resolution occurred in 72%, 77%, and 87% of patients treated with rivaroxaban and in 48%, 68%, and 80% of those treated with warfarin, respectively (*P* = 0.084). No ischemic strokes occurred in the rivaroxaban group, compared with 4 events in the warfarin group (*P* = 0.08). These findings should be interpreted cautiously, given the limited sample size, open-label design, and the fact that only 50% of participants were receiving dual antiplatelet therapy, limiting applicability to patients with recent coronary stent implantation.

More recently, the RIVAWAR trial randomized 261 patients with acute coronary syndromes, 90% of whom presented with STEMI, to receive rivaroxaban or warfarin in combination with standard antiplatelet therapy.^8^ At 3 months, no significant differences were observed in thrombus resolution, mortality, ischemic stroke, or major bleeding between treatment groups. Despite being one of the largest randomized studies addressing anticoagulation in this context, RIVAWAR was limited by its single-center design, open-label methodology, and lack of long-term follow-up, particularly in high-risk populations such as patients with type 4b myocardial infarction, as in the present case.^8^ Importantly, although prospective randomized evidence is currently limited to rivaroxaban-based regimens, available observational data and meta-analyses suggest comparable effectiveness among different direct oral anticoagulants, supporting consideration of DOACs as a class alternative to vitamin K antagonists rather than preferential use of a specific agent.^6^

Given these limitations, extrapolating data from randomized trials evaluating anticoagulant-based strategies in combination with antiplatelet therapy for other indications remains challenging.^9^^,^^10^ Although these studies consistently demonstrated reduced bleeding with DOAC-based regimens compared with vitamin K antagonist–based triple therapy, they were generally underpowered to detect definitive differences in thrombotic outcomes. Consequently, the optimal duration of triple antithrombotic therapy in patients with concomitant stent thrombosis and LVT remains uncertain. While shorter durations are commonly favored to mitigate bleeding risk, selected patients with very high thrombotic risk and low bleeding risk may reasonably be considered for prolonged triple therapy beyond 1 month, based on individualized assessment. In high-risk scenarios such as concomitant stent thrombosis and LVT, the clinical priority therefore shifts toward mitigation of embolic risk through a carefully individualized approach.

## Conclusions

**Figure 3:**
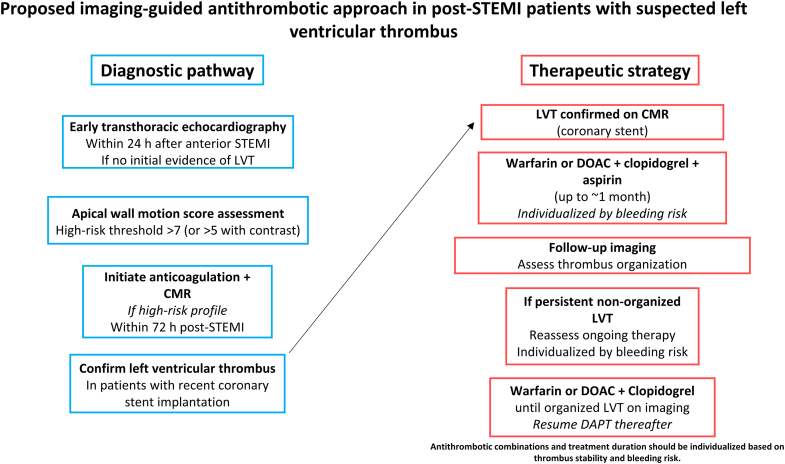
Imaging-Guided Approach to the Diagnosis and Management of LVT After Anterior STEMI Profound apical dysfunction on TTE prompts anticoagulation and confirmation with CMR. In patients with recent coronary stent implantation, antithrombotic therapy is dynamically adjusted based on thrombus persistence or organization on follow-up imaging. CMR = cardiac magnetic resonance; DAPT = dual antiplatelet therapy; DOAC = direct oral anticoagulant; LVT = left ventricular thrombus; STEMI = ST-segment elevation myocardial infarction; TTE = transthoracic echocardiography.

This case highlights the complexity of antithrombotic management when LVT complicates anterior STEMI in the setting of concomitant coronary stent thrombosis. In this patient, delayed and ineffective reperfusion, extensive anterior myocardial injury, and severely reduced left ventricular ejection fraction created a pronounced thrombotic milieu. In the absence of medication nonadherence or technical stent failure, priority was given to mitigating thromboembolic risk through short-term triple antithrombotic therapy. Profound apical dysfunction on echocardiography prompted early anticoagulation and confirmatory CMR, enabling dynamic, imaging-guided reassessment of antithrombotic therapy (Figure 3). Such an individualized approach may help balance thrombotic and bleeding risks in selected high-risk post-STEMI patients, underscoring the need for further evidence to define the optimal anticoagulation regimen for patients with LVT, particularly in those requiring concomitant antiplatelet therapy.

The absence of postdischarge follow-up represents an important limitation, as it precludes evaluation of thrombus resolution, bleeding events, and long-term clinical outcomes. Nevertheless, this case provides relevant insights into imaging-guided decision-making in a complex thrombotic scenario with limited evidence.

### Ethics Statement

Written informed consent was obtained from the patient for the publication of this case report and accompanying images.

## Funding Support and Author Disclosures

This work was supported by the Fundación Universitaria Sanitas and the Vice Presidency of Innovation and Scientific Development, Clínica Colsanitas S.A., Grupo Keralty, Bogotá, Colombia. The authors have reported that they have no relationships relevant to the contents of this paper to disclose.
